# Proteomic profiling of cerebrospinal fluid in pediatric myelin oligodendrocyte glycoprotein antibody-associated disease

**DOI:** 10.1007/s12519-022-00661-y

**Published:** 2022-12-12

**Authors:** Yi-Long Wang, Meng-Ying Zhu, Zhe-Feng Yuan, Xiao-Yan Ren, Xiao-Tong Guo, Yi Hua, Lu Xu, Cong-Ying Zhao, Li-Hua Jiang, Xin Zhang, Guo-Xia Sheng, Pei-Fang Jiang, Zheng-Yan Zhao, Feng Gao

**Affiliations:** 1 Department of Neurology, Children’s Hospital, Zhejiang University School of Medicine, Hangzhou 310052, China; 2 Children’s Hospital, Zhejiang University School of Medicine, Hangzhou 310052, China; 3 Children’s Hospital, National Clinical Research Center for Child Health, Zhejiang University School of Medicine, Hangzhou 310052, China

**Keywords:** Acute disseminated encephalomyelitis, Cerebrospinal fluid, Complement cascades, Myelin oligodendrocyte glycoprotein-associated disease, Proteomics

## Abstract

**Background:**

Myelin oligodendrocyte glycoprotein (MOG) antibody-associated disease (MOGAD) is an autoimmune demyelinating disorder of the central nervous system.

**Methods:**

Extracted proteins from 34 cerebrospinal fluid (CSF) samples [patients with MOGAD (MOG group, *n* = 12); healthy controls (HC group, *n* = 12); patients with MOG seronegative and metagenomics next-generation sequencing-negative inflammatory neurological diseases (IND group, *n* = 10)] were processed and subjected to label-free quantitative proteomics. Supervised partial least squares-discriminant analysis (PLS-DA) and orthogonal PLS-DA (O-PLS-DA) models were also performed based on proteomics data. Functional analysis of differentially expressed proteins (DEPs) was performed using Gene Ontology, InterPro, and Kyoto Encyclopedia Genes and Genomes. An enzyme-linked immunosorbent assay was used to determine the complement levels in serum from patients with MOGAD.

**Results:**

Four hundred and twenty-nine DEPs (149 upregulated and 280 downregulated proteins) were identified in the MOG group compared to the HC group according to the *P* value and fold change (FC). Using the O-PLS-DA model, 872 differentially abundant proteins were identified with variable importance projection (VIP) scores > 1. Five proteins (gamma-glutamyl hydrolase, cathepsin F, interalpha-trypsin inhibitor heavy chain 5, latent transforming growth factor beta-binding protein 4 and leukocyte-associated immunoglobulin-like receptor 1) overlapping between the top 30 DEPs with top-ranked *P* value and FC and top 30 proteins in PLS-DA VIP lists were acquired. Functional analysis revealed that the dysregulated proteins in the MOG group were primarily involved in complement and coagulation cascades, cell adhesion, axon guidance, and glycosphingolipid biosynthesis compared to the HC group.

**Conclusion:**

The proteomic alterations in CSF samples from children with MOGAD identified in the current study might provide opportunities for developing novel biomarker candidates.

**Supplementary Information:**

The online version contains supplementary material available at 10.1007/s12519-022-00661-y.

## Introduction

Myelin oligodendrocyte glycoprotein (MOG) is an immunoglobulin protein uniquely expressed on the plasma membrane of oligodendrocyte cells and the outer surface of myelin sheaths, consisting of 218 amino acids with a size of 28 kDa [1, 2]. MOG antibody (Ab)-associated disease (MOGAD) is an autoimmune demyelination disorder of the central nervous system (CNS) characterized by the emergence of Abs against MOG [3–5]. Patients with MOGAD usually have clinical phenotypes that demonstrate some overlap among several CNS acquired demyelinating syndromes, such as acute disseminated encephalomyelitis (ADEM), optic neuritis (ON), myelitis, encephalitis, aquaporin-4 (AQP4) Ab-negative neuromyelitis optica spectrum disorder (NMOSD), and others [5, 6]. The clinical phenotype changes with age from opticospinal (ON, myelitis, and brainstem encephalitis) in adults to ADEM-like (ADEM, ADEM-ON and encephalitis) in children [7–10]. The diagnosis of MOGAD based on the combination of clinical features and neuroimaging findings lacks specificity. Therefore, appropriate Ab testing is crucial for MOGAD diagnosis. Traditional testing methods, such as immunohistochemistry, enzyme-linked immunosorbent assay (ELISA) or Western blotting, are unsatisfactory because of their low sensitivity. Over the past decade, a great deal of effort has been made to improve MOG-Ab detection techniques [5]. Cell-based assay (CBA) is currently the gold standard method for Ab testing with full-length human MOG [11]. While the timing of sampling and testing is important, as Ab titers shift and might decline several months after initial presentation, some patients even subsequently acquired negative test results [10]. Different cutoff values of seropositivity also influence the sensitivity and specificity of testing results [12]. In a large multicenter study, CBA of MOG Abs yielded good agreement in high positive and negative samples; however, the agreement was decreased in the low positive or borderline samples [13]. Despite refinements in the accuracy of MOG-Ab testing assays, a proportion of patients might miss early diagnosis based on accurate detection of MOG Abs and suffer from poor prognosis. Thus, identifying novel specific biomarkers of MOGAD is necessary for diagnostic and treatment purposes.

Proteomics is the comprehensive study of proteins and their identification, localization, biological structure, and function [14]. Mass spectrometry (MS)-based proteomics has provided unbiased identification and quantification of untargeted proteins. MS has become a valuable tool for several chemical and biological molecule investigations, especially for biological biomarker detection [15–19]. In recent studies, MS-based proteomics has demonstrated great promise in identifying cerebrospinal fluid (CSF) biomarkers for CNS autoimmune disease and neurodegenerative disease [20–26]. MOG Abs were measured through CBA in serum samples because they are present in the CSF at low levels. Here, we applied proteomics to analyze the expression characteristics of proteins in CSF from patients with MOGAD for a comprehensive search for novel biomarkers.

In the present study, we characterized the protein expression profiles in CSF samples from children with MOGAD compared with healthy controls (HCs) or children with MOG seronegative and metagenomics next-generation sequencing (mNGS)-negative inflammatory neurological diseases (INDs) using an MS-based proteomics method. Here, we identified several novel protein markers in CSF that could contribute to the diagnosis of MOGAD, and our results might also help researchers study the molecular mechanism of MOAGD and facilitate progress in therapy plans.

## Methods

### Study outline and patient characteristics

The study was approved by the Ethics Committee of Children’s Hospital, Zhejiang University School of Medicine (2021-IRB-161). Written informed consent was obtained from the guardians. Twelve pediatric patients with MOGAD were diagnosed in the department of neurology at the hospital. Ten children with MOG seronegative and mNGS-negative IND with normal white blood cell (WBC) counts in CSF and magnetic resonance imaging (MRI) results were also recruited for the study. Children with vascular headache were enrolled as controls, and they were both age- and gender-matched with children in the MOGAD group. The diagnosis of MOGAD was based on the combination of comprehensive clinical assessments, laboratory Ab testing results and brain MRI scan results according to the criteria of Jarius et al. [11]. All pediatric patients with MOGAD were positive for MOG Abs and negative for AQP4 Abs using a commercial fixed CBA (Euroimmun, Lubeck, Germany). The initial and final diagnoses of the cases, including ADEM, ADEM-ON, ON, transverse myelitis (TM) and encephalitis, were based on the most recent diagnostic criteria for these diseases [5, 7]. Clinical data were collected from electronic medical records.

### Sample collection

CSF was obtained through a lumbar puncture within 24 hours after admission. Briefly, a needle was inserted between the lumbar 3 and 4 (L3–L4) lumbar vertebrae, and 0.3–0.8 mL of collected CSF was immediately stored at 4 °C in the refrigerator and centrifuged at 4 °C for 10 minutes at 1000 ×g for 2 hours. Then, after centrifugation, the samples were stored at − 80 °C. The remaining 2–3 mL CSF samples were sent to clinical laboratories for routine biochemical analysis. All blood-stained CSF samples were excluded from this study.

### Extraction and quantification of cerebrospinal fluid proteins

CSF specimens were thawed and transferred into a 1.5 mL centrifuge tube and lysed with DB lysis buffer [8 M urea (10023218, Sinopharm) and 100 mM triethylammonium bicarbonate (TEAB; T7408-500ML, Sigma, pH 8.5)]. The lysate was centrifuged at 12,000 × g for 15 minutes at 4 °C. The supernatant was deoxidized using 10 mM DL-dithiothreitol (D9163-25G, Sigma) for 1 hour at 56 °C, followed by alkylation with adequate iodoacetamide (I6125-25G, Sigma) for 1 hour at room temperature in the dark.

A linear protein concentration ranging from 0 to 0.5 g/L was prepared as bovine serum albumin (BSA) standard protein solution following the Bradford protein quantitative kit (P0006, Beyotime) manual. The sample solutions with gradient dilutions and BSA standard protein solutions in a 20 μL volume were placed into a 96-well plate. The plate was filled with 180 μL of G250 dye solution immediately and stored at room temperature, and the absorbance of each well was determined at 595 nm after 5 minutes of incubation. The absorbance of the standard protein solution was used to create the standard curve, and subsequently, the concentration of the protein sample was calculated. Each assay was conducted in triplicate. Equal amounts of the protein sample (20 μg) were separated by 12% SDS‒PAGE. The gel was dyed with Coomassie brilliant blue R-250 and decolored until the bands were visualized clearly.

### Cerebrospinal fluid protein digestion

DB lysis buffer (8 M urea, 100 mM TEAB, pH 8.5), trypsin, and 100 mM TEAB were added to each protein sample, and the mixture was digested at 37 °C for 4 hours. Then, trypsin and CaCl_2_ were added for digestion overnight. Formic acid (FA; A117-50, Thermo Fisher Scientific) was added to the digested sample to adjust the pH to below three, and the digested sample was centrifuged at 12,000 × g for 5 minutes at room temperature. The supernatant was gradually transferred to a C18 desalting column. Washing buffer [0.1% FA, 3% acetonitrile (A955-4, Thermo Fisher Chemical)] was used to wash the column thrice, and elution buffer (0.1% FA, 70% acetonitrile) was loaded onto the column. The elution of each sample was collected and freeze-dried [27].

### Data-dependent acquisition (DDA) spectrum library construction

#### Separation of peptides

A gradient elution (a mixture of mobile phases), composed of mobile phase A [2% acetonitrile, pH 10.0 adjusted with ammonium hydroxide (221228-500ML-A, Sigma)] and B (98% acetonitrile, pH 10.0 adjusted with ammonium hydroxide), was prepared. The lyophilized protein powder obtained was dissolved in solution A and then centrifuged at 12,000 × g for 10 minutes at room temperature. The resulting products were fractionated by a Rigol L3000 HPLC system using a C18 column (Waters BEH C18, 4.6 × 250 mm^2^, 5 μm), and the column oven temperature was set at 45 °C. The detailed elution gradient procedure is shown in Supplementary Table 1. The column eluates were monitored using ultraviolet absorbance at 214 nm and collected into a fresh tube at one-minute intervals. Finally, these were combined into four fractions and dried using a vacuum concentrator. The purified peptides were reconstituted in 0.1% (*v*/*v*) FA in water. The peptides of twelve MOGAD samples, ten IND samples and twelve HC samples were pooled and lyophilized before liquid chromatography-mass spectrometry (LC‒MS) analysis.

#### LC‒MS/MS analysis for data-dependent acquisition mode

Shotgun proteomics analyses for transition library construction were performed on a Thermo Fisher Q ExactiveTM HF-X MS coupled with an Evosep One UHPLC system (Evosep). Data were acquired using the DDA mode. Four micrograms of peptide supernatant was mixed with 0.8 μL of iRT (indexed retention time) calibration peptides (Biognosys), loaded on a 15 cm-long column with a 150 μm inner diameter, and packed in-house with 1.9 μm C18 beads. Elution was achieved using a linear gradient, as demonstrated in Supplementary Table 2. The resulting peptides were analyzed using the Thermo Fisher Q ExactiveTM HF-X MS. Ionization was conducted using a Nanospray Flex^™^ (electrospray ionization) with a spray voltage of 2.1 kV and a capillary temperature of 320 °C. A full scan with a mass ranging from 350 to 1500 mass/charge ratio (m/z) was performed at 120,000 resolution (at 200 m/z) with an automatic gain control (AGC) setting of 3 × 10^6^ and a maximum ion injection time of 80 ms. The top 40 most abundant precursor ions were selected in each MS scan and sequentially fragmented by higher-energy collisional dissociation (HCD), followed by MS/MS analysis with a resolution of 17,500 at 200 m/z (27% collision energy, 5 × 10^4^ AGC target, 45 ms maximum ion time). The intensity threshold was set at 1.1 × 10^4^, and the dynamic exclusion was set at 20 seconds. The raw data obtained from MS detection were deposited as “raw” and used to generate the DDA spectrum library.

#### LC‒MS/MS analysis-data-independent acquisition (DIA) mode

A gradient elution composed of mobile phases A (0.1% FA in water) and B (0.1% FA in 80% acrylonitrile) was developed. Four micrograms of peptide supernatant was mixed with 0.8 μL iRT calibration peptides and then analyzed using a Thermo Fisher Q ExactiveTM HF-X mass spectrometer coupled with an Evosep One UHPLC system (Evosep) in DIA mode. Ionization was conducted on a Nanospray Flex^™^ (electrospray ionization) with a spray voltage of 2.1 kV and a capillary temperature of 320 °C. DIA was achieved with a mass range of 350–1500 m/z at 60,000 resolution (at 200 m/z) and an ACG setting of 5 × 10^5^, and the maximum ion injection time was 20 ms. The peptides were sequentially fragmented by HCD, followed by MS2 analysis with a resolution of 30,000 at 200 m/z (27% collision energy, 1 × 10^6^ AGC target). The detailed scan window information of DIA mode is listed in Supplementary Table 3. The raw data obtained from MS detection were deposited as “raw” and used to generate the DIA spectrum library.

#### The identification and quantitation of protein

The MS/MS spectra were searched separately against homo_sapiens_uniprot_2021_7_15. A database search was performed using Proteome Discoverer 2.2 (PD 2.2, Thermo Fisher Scientific) with Fasta (202195 sequences). Data search parameters were set with precursor mass tolerance of product ion and precursor of 10 ppm 0.02 Da, respectively. Carbamidomethyl (C) was set as a fixed modification, whereas oxidation of methionine (M) was set as a dynamic modification. N-terminal acetylation was set as a modification in PD 2.2. Trypsin was selected for cleavage specificity, and a maximum of two missed cleavage peptides was allowed. The credibility of peptide spectrum matches (PSMs) was set to more than 99% for PSM identification using the software PD 2.2 to further improve the quality of the analysis data. Additionally, we required at least one unique peptide for protein identification. The false discovery rate threshold was set at 1.0% for identifying PSMs and proteins. Peptide search and identification were performed using PD 2.2, and the acquired raw data were analyzed using Spectronaut (version 14.0, Biognosys) software to construct a library. To create a target list, the qualified peptides and product ions were selected from the resulting spectra under peptide and ion-pair selection criteria. The ion-pair chromatographic peaks were obtained after importing DIA data following the target list. The calculated peak area and the ion were matched to obtain qualitative and quantitative properties of peptides. The protein quantification results were analyzed by performing Student’s *t* test. The iRT calibration peptides were spiked into the sample to correct the retention time. The Q value cutoff for the precursor ion was set at 0.01.

#### Serum complement detection

Blood samples were collected in ethylenediaminetetraacetic acid-containing tubes via peripheral venipuncture. These tubes were placed at room temperature for 30 minutes for clot retraction, followed by centrifugation at 4 °C. The final serum samples were obtained and stored at − 80 °C until analysis. C1r, C2, C5 and C9 in serum from patients with MOGAD and HCs were detected using corresponding ELISA kits (Cloud-Clone Corp, Wuhan, China) according to the manufacturer’s recommendations.

#### Data analysis

As a pre-processing dimension reduction method, principal component analysis (PCA) was used to compare the protein content of each sample. Fold change (FC) is defined as the ratio of the average value of all biological replicated quantitative values of each protein between each pair of samples. We first examined differentially expressed proteins (DEPs) between the experimental and control groups based on an FC criterion of > 1.5 or ≤ 0.67 and a *P* < 0.05. A heatmap was generated to visualize the DEPs using the R packages pheatmap and ggplot2. Subsequently, supervised partial least squares-discriminant analysis (PLS-DA) and orthogonal PLS-DA (O-PLS-DA) were performed using scripts written in R language (Ropls package) to determine the potential of a specific protein biomarker. The analysis of variable importance projection (VIP) score was used to assess the relative magnitude of the observed changes between the experimental and control groups. We considered proteins with VIP values of 1 and above as the best classifiers. Finally, the proteins overlapping between the top 30 DEPs (identified by FC > 1.5 or ≤ 0.67 and *P* < 0.05) and top 30 proteins in the O-PLS-DA VIP lists were selected as the candidate biomarkers.

#### Bioinformatics and pathway analysis

Gene Ontology (GO) and InterPro (IPR) functional analyses were conducted using the interproscan program against the non-redundant protein database (including Pfam, PRINTS, ProDom, SMART, ProSite, PANTHER) [28]. Kyoto Encyclopedia Genes and Genomes (KEGG) analysis was conducted to analyze the protein families and pathways [29]. DEPs were used for GO, IPR and KEGG enrichment analyses (Supplementary Fig. 1).

## Results

### Clinical features of participants

A total of 34 children were enrolled in this study, including twelve children with MOGAD, ten children with IND (MOG seronegative and mNGS-negative patients), and twelve HCs. The demographic data of all participants with MOGAD are summarized in Table 1. The mean age of the disease onset of children with MOGAD was 9.12 ± 3.01 years. Seven (58.3%) patients with MOGAD were female, and five (41.7%) were male. The clinical phenotypes of MOGAD patients enrolled in the study were ADEM in six (60%) cases, encephalitis in four (33.3%) cases, ADEM-ON in one (8.3%) case and ON in one (8.3%) case (Table 1). Ten patients in the IND group consisted of seven female and three male children with a mean age of 6.49 ± 3.79 years. The clinical phenotypes of patients in the IND group included in the study were ADEM in four (40%) cases, encephalitis in five (50%) cases, and ON in one (10%) case (Table 2).

**Table 1:** Clinical features and serological findings of MOGAD group at diagnosis

Case no.	Age at onset (y)/sex	Anti-MOG Ab titer (serum)	Anti-AQP4 Ab titer (serum)	Therapeutics	Clinical phenotype	CSF OB
1	5.83/M	+ 1:10	Negative	GCs, IVIG, ACV	ADEM	Negative
2	8.08/M	++ 1:32	Negative	GCs, IVIG, ACV	EN	Negative
3	11.17/F	+ 1:10	Negative	GCs, IVIG, ACV	EN	Negative
4	10.5/M	++ 1:32	Negative	GCs, IVIG, ACV, AZT	ADEM + ON	Negative
5	5.67/F	++ 1:100	Negative	GCs, IVIG, ACV	ON	Negative
6	12.92/F	+ 1:10	Negative	GCs, IVIG, MMF, ACV	ADEM	Negative
7	5.58/F	+ 1:10	Negative	GCs, IVIG, ACV	ADEM	Negative
8	13.25/M	++ 1:100	Negative	GCs, IVIG, ACV	EN	Negative
9	12.42/F	++ 1:32	Negative	GCs, ACV	ADEM	Negative
10	8.83/M	++ 1:100	Negative	GCs, ACV	ADEM	Positive
11	5.33/F	++ 1:100	Negative	GCs, ACV	EN	Negative
12	9.92/F	+ 1:10	Negative	GCs, ACV, IVIG	ADEM	Negative

*MOG* myelin oligodendrocyte glycoprotein, *AQP4* aquaporin-4, *MOGAD* MOG antibody-associated disease, *M* male, *F* female, *GCs* glucocorticoids, *ACV* acyclovir, *MMF* mycophenolate mofetil, *AZT* azathioprine, *IVIG* intravenous immunoglobulin, *ADEM* acute disseminated demyelinating syndromes, *ON* optic neuritis, *EN* encephalitis. *Ab* antibody, *CSF* cerebrospinal fluid, *OB* oligoclonal band. “ + ” weakly positive, “ ++ ” positive

**Table 2:** Clinical features and serological findings of IND group at diagnosis

Case no	Age at onset (y)/sex	Antibodies titer (serum)			mNGS	Therapeutics	Clinical phenotype	CSF OB
		Anti-MOG Ab	Anti-AQP4 Ab	Anti-MBP Ab				
1	2.58/F	Negative	Negative	Negative	Negative	GCs	ADEM	Negative
2	11.33/F	Negative	Negative	Negative	Negative	GCs, ACV, IVIG	ADEM	Negative
3	7.33/F	Negative	Negative	Negative	Negative	GCs, ACV, IVIG	ADEM	Negative
4	2.08/F	Negative	Negative	Negative	Negative	ACV, IVIG	EN	Negative
5	9.00/F	Negative	Negative	Negative	Negative	None	EN	Negative
6	2.08/F	Negative	Negative	Negative	Negative	GCs, ACV	EN	Positive
7	13.00/M	Negative	Negative	Negative	Negative	GCs, ACV	ADEM	Negative
8	6.58/F	Negative	Negative	Negative	Negative	GCs	EN	Negative
9	5.67/M	Negative	Negative	Negative	Negative	GCs, ACV	EN	Negative
10	5.25/M	Negative	Negative	Negative	Negative	GCs, ACV	ON	Negative

*IND* myelin oligodendrocyte glycoprotein antibody-seronegative and metagenomics next-generation sequencing-negative inflammatory neurological diseases, *MOG* myelin oligodendrocyte glycoprotein, *AQP4* aquaporin-4, *MBP* myelin basic protein, *M* male, *F* female, *Ab* antibody, *mNGS* metagenomics next-generation sequencing, *CSF* cerebrospinal fluid, *OB* oligoclonal band, *GCs* glucocorticoids, *ACV* acyclovir, *IVIG* intravenous immunoglobulin, *ADEM* acute disseminated encephalomyelitis, *ON* optic neuritis, *EN* encephalitis

### Clinical features and laboratory findings of patients with MOGAD and patients with IND

The cranial and spinal MRIs of cases and the orbital MRIs of patients with ON were conducted. The reports were verified by a senior radiologist and neurologist. Typical MRI findings (widespread supra- and infratentorial, asymmetrical diffuse white matter T2-hyperintensive lesions) of ADEM were observed in seven cases, and three cases had spinal cord involvement. Cranial MRIs of one case with ON were normal, and orbital MRIs demonstrated bilateral optic nerve thickening (Table 3). Representative cranial and spinal MRIs of the study group are shown in Supplementary Fig. 2.

**Table 3:** Cerebrospinal fluid findings and radiological of MOGAD group at diagnosis

Case no	CSF pleocytosis (WBC 106 cells per μL)	CSF protein (normal < 0.45 g/L)	CSF glucose (normal 2.78–4.5 nmol/L)	Lesion location in MRI at disease onset
1	5	0.18	4.30	Bilateral cerebral hemisphere and thalamus
2	12	0.23	4.68	Bilateral thalamus
3	5	0.11	3.18	No lesions
4	2	0.35	4.61	Bilateral frontal lobe, parietal lobe
5	6	0.24	3.66	Bilateral neuritis optica
6	284	0.62	3.88	Bilateral thalamus, bilateral frontal lobe, myelitis
7	32	0.35	4.67	Diffuse asymmetric white matter, myelitis
8	20	0.17	4.54	Right frontal lobe
9	73	0.76	2.91	Diffuse asymmetric white matter, myelitis
10	23	0.25	3.38	Diffuse asymmetric white matter
11	143	0.45	3.33	Right thalamus
12	9	0.26	4.10	Multiple (bilateral cerebral hemisphere, cerebellum, right basal ganglia, brain stem), myelitis

*CSF* cerebrospinal fluid, *MOGAD* myelin oligodendrocyte glycoprotein antibody-associated disease, *MRI* magnetic resonance imaging, *WBC* white blood cell

CSF sampling was performed at an initial presentation on all patients with MOGAD. The median WBC count in the CSF of patients with MOGAD was 51 cells/μL (range: 2–284 cells/μL), and seven of twelve patients had pleocytosis in the CSF. Almost all patients with MOGAD had normal CSF protein concentrations, except two cases (cases 6 and 9) (normal reference range: < 0.45 g/L), with a median concentration of 0.33 g/L (range: 0.11–0.76 g/L). The median concentration of CSF glucose concentrations of twelve patients with MOGAD was 3.94 mmol/L (range: 2.91–4.68 mmol/L; normal reference range: 2.78–4.5 mmol/L) (Table 3).

The brain and spinal MRI findings and other clinical features in patients with IND are summarized in Table 2. The median WBC count in the CSF of cases in the IND group was 21 cells/μL (range: 2–284 cells/μL), and four of ten patients had pleocytosis in the CSF. All patients in the IND group had normal CSF protein concentrations (normal reference range: < 0.45 g/L), with a median concentration of 0.24 g/L (range: 0.14–0.40 g/L). The median concentration of CSF glucose concentrations of ten patients in the IND group was 4.57 mmol/L (range: 3.54–6.95 mmol/L; normal reference range: 2.78–4.5 mmol/L) (Table 4).

**Table 4:** Cerebrospinal fluid findings and radiological of IND group at diagnosis

Case no	CSF pleocytosis (WBC 106 cells per μL)	CSF protein (normal < 0.45 g/L)	CSF glucose (normal 2.78–4.5 nmol/L)	Lesion location in MRI at disease onset
1	2	0.19	5.70	Bilateral frontal lobe
2	34	0.30	6.95	Myelitis
3	97	0.40	4.63	No lesions
4	1	0.20	3.79	Left basal ganglia, left anterior and temporal horn
5	24	0.30	3.75	Diffuse asymmetric white matter
6	2	0.19	3.54	Diffuse asymmetric white matter, myelitis
7	45	0.23	4.51	Diffuse asymmetric white matter, myelitis
8	1	0.14	3.80	No lesions
9	1	0.29	5.36	Bilateral cerebellar hemisphere, myelitis
10	2	0.15	3.69	No lesions

*CSF* cerebrospinal fluid, *IND* myelin oligodendrocyte glycoprotein antibody-seronegative and metagenomics next-generation sequencing-negative inflammatory neurological diseases, *MRI* magnetic resonance imaging

### Modulation in the proteome pattern in the CSF of MOGAD patients and HCs

**Fig. 1:**
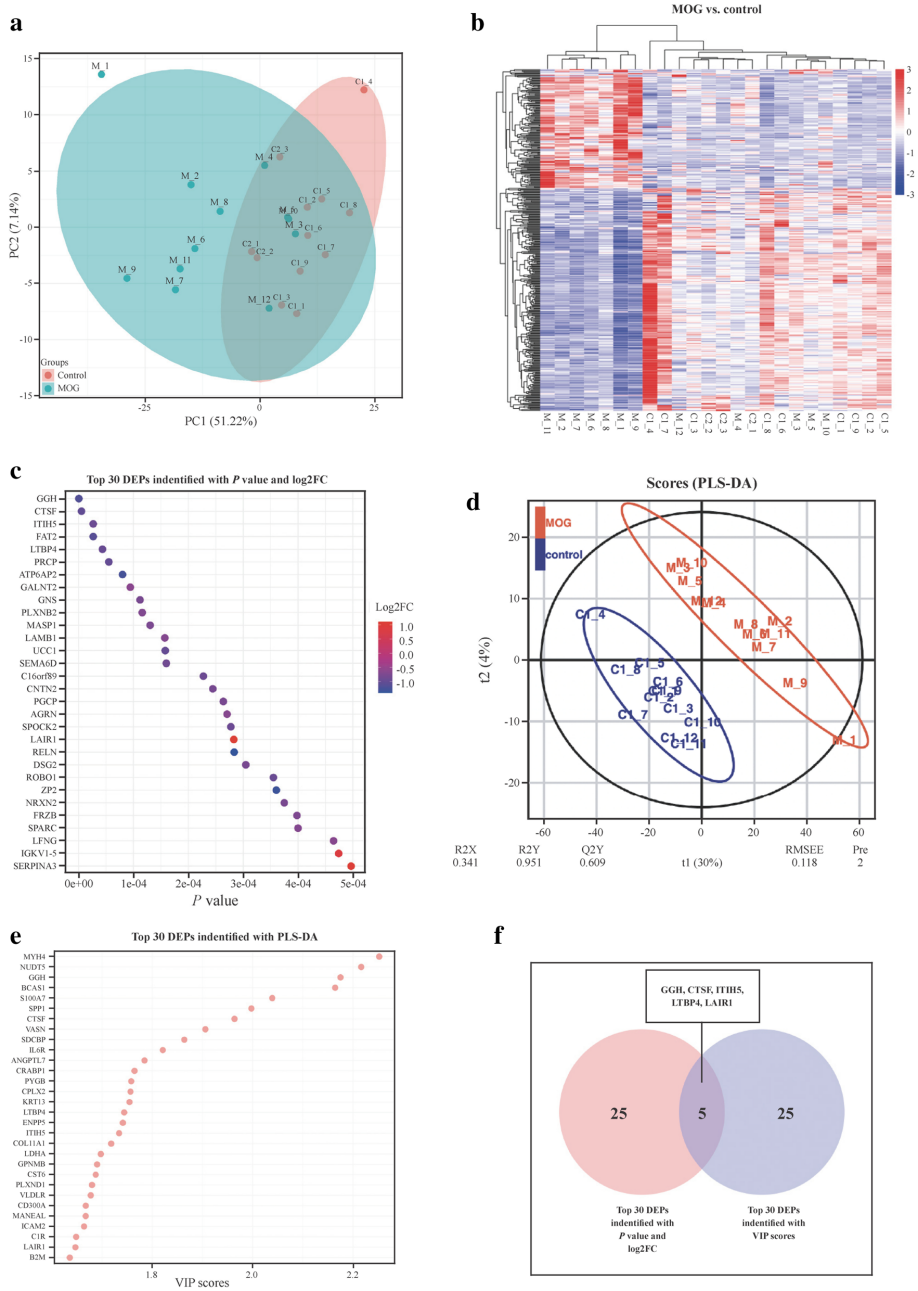
Characteristics of DEPs between children with MOGAD and healthy children. **a** PCA of whole proteins from proteomics data; **b** heatmap of cluster analysis based on MS results; **c** top 30 DEPs identified with *P* value and FC; **d** O-PLS-DA of DEPs between children with MOGAD and healthy children; **e** top 30 DEPs identified with most importance using O-PLS-DA; **f** five DEPs selected with top-ranked *P* value, FC and VIP scores. *DEPs* differentially expressed proteins, *MOGAD* myelin oligodendrocyte glycoprotein antibody-associated disease, *PCA* principal component analysis, *MS* mass spectrometry, *O-PLS-DA* orthogonal partial least squares-discriminant analysis, *FC* fold change, *VIP* variable importance projection

We performed PCA on the CSF proteomics data to visualize the overall trend in protein expression levels among all samples (Fig. 1a). The preliminary examination revealed that protein expression levels differed between MOGAD and HCs. To investigate the remarkable changes in CSF proteins between MOGAD patients and HCs, DIA with high data completeness was used to perform proteomic analysis on CSF samples of MOGAD children and HCs [30]. A total of 1517 proteins (149 upregulated and 280 downregulated proteins) were identified and quantified with FC > 1.5 or ≤ 0.67 and *P* < 0.05 in the CSF from the MOGAD group compared with HCs (Fig. 1b). The top 30 DEPs with the top-ranked *P* value and FC are shown in Fig. 1c.

In consideration of the model fit and predictability, PLS-DA and O-PLS-DA were subsequently implemented to reduce data dimensionality (Fig. 1d). Total variation with two components that separated MOG patients and HCs is clear in Fig. 1d (R2X = 0.341, and R2Y = 0.951, Q2Y = 0.609), demonstrating that CSF proteome change holds value in the identification of MOG patients from HCs. In the O-PLS-DA model, the corresponding VIP score was calculated to assess the identification performance of the investigated protein. Eight hundred seventy-two differentially abundant proteins were identified with VIP scores > 1. The top 30 proteins with the highest VIP scores in O-PLS-DA are displayed in Fig. 1e.

Finally, five proteins [gamma-glutamyl hydrolase (GGH), cathepsin F (CTSF), interalpha-trypsin inhibitor heavy chain 5 (ITIH5), latent transforming growth factor beta-binding protein 4 (LTBP4) and leukocyte-associated immunoglobulin-like receptor 1 (LAIR1)] overlapping between the top 30 DEPs with top-ranked *P* value and FC and top 30 proteins in O-PLS-DA VIP lists were selected as the candidate biomarkers and presented in Fig. 1f. GGH, a critical enzyme in maintaining folate homeostasis through catalyzing hydrolysis, acts as the most important variable to differentiate MOG patients from HCs. It has been demonstrated that folate intake during pregnancy is important in myelin maintenance [31]. CTSF is also among the top five most important variables listed to discriminate patients. Previous studies revealed that inhibitors of this enzyme might be useful in treating certain diseases associated with inappropriate or excessive immune responses [32].

**Fig. 2:**
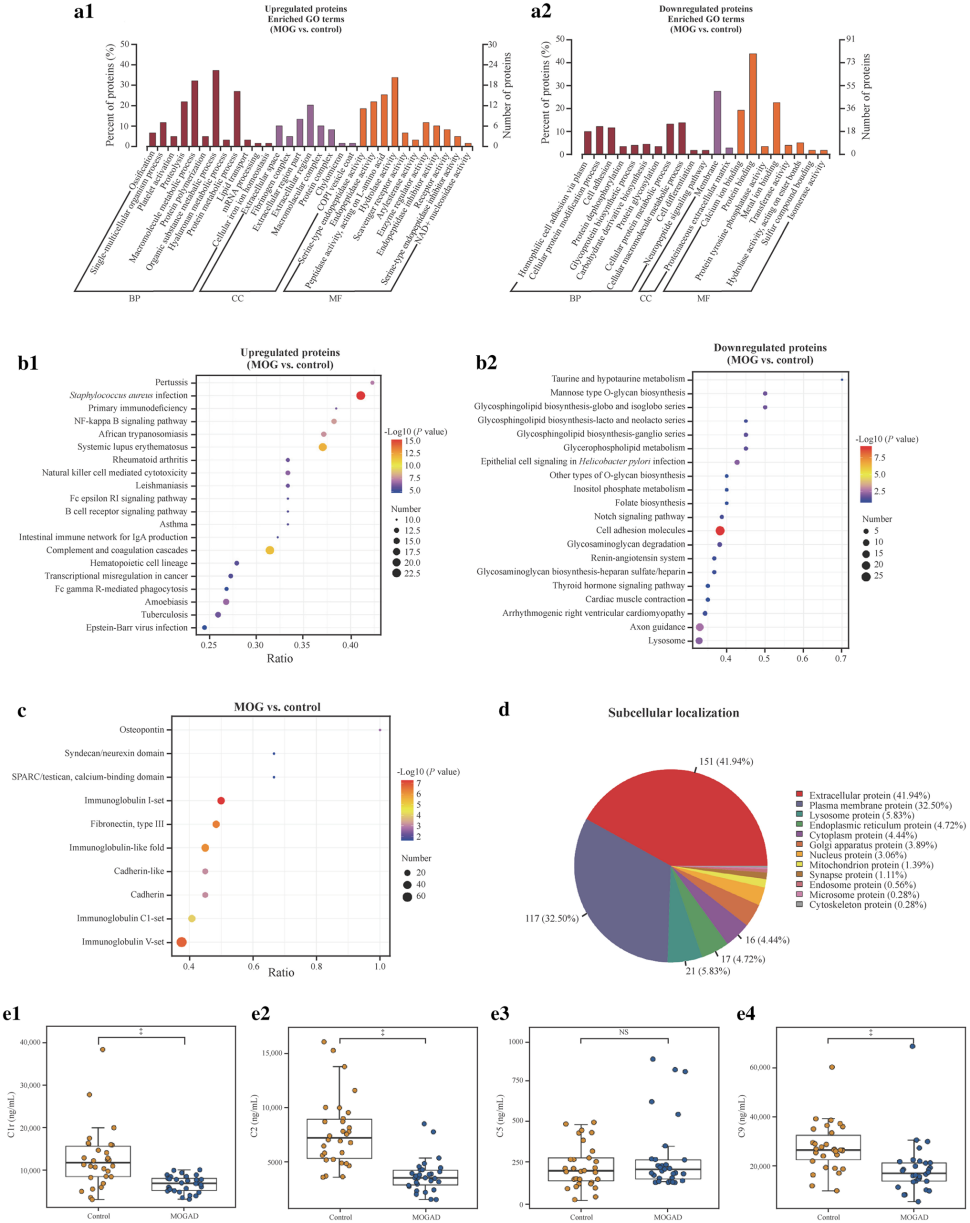
Functional analysis of DEPs between children with MOGAD and healthy children. **a** GO terms analysis on upregulated (**a1**) and downregulated proteins (**a2**) in MOGAD children compared with IND children are listed with lengths of the bars representing the numbers of related DEPs as labeled at the bottom; **b** the top 20 KEGG analysis for upregulated (**b1**) and downregulated (**b2**) are demonstrated pathways with the size of dots representing the numbers of related DEPs displayed at the bottom; **c** IPR analysis of DEPs: distribution of the DEPs in MOGAD versus IND comparison with IPR annotation; **d** functional classification by subcellular localization; **e** complement protein levels in serum from children with MOGAD compared with HCs. ^‡^*P* < 0.001 (Mann‒Whitney *U* test). *DEPs* differentially expressed proteins, *MOGAD* myelin oligodendrocyte glycoprotein antibody-associated disease, *IND* MOG seronegative and metagenomics next-generation sequencing-negative inflammatory neurological diseases, *HCs* healthy controls, *GO* gene ontology, *KEGG* Kyoto Encyclopedia Genes and Genomes, *IPR* InterPro, *NS* not significant, *BP* biological process, *CC* cellular component, *MF* molecular function

We then performed GO term, KEGG, IPR, and subcellular localization analyses to investigate the potential functions of the DEPs determined by FC > 1.5 or ≤ 0.67 and *P* < 0.05. In the GO enrichment analysis, the upregulated proteins were primarily enriched in single-multicellular organism process, ossification, platelet activation, protein polymerization, and proteolysis (Fig. 2a1). In contrast, the downregulated proteins were mainly related to homophilic cell adhesion via plasma membrane adhesion molecules, cellular protein modification processes, cell adhesion, protein dephosphorylation, glycoprotein biosynthetic processes and protein glycosylation (Fig. 2a2). In the KEGG pathway analysis, the upregulated proteins were principally related to complement and coagulation cascades, the Toll-like receptor signaling pathway, and the NF-kappa B signaling pathway (Fig. 2b1), whereas the downregulated proteins were primarily enriched in cell adhesion molecules, axon guidance, lysosomes, glycosphingolipid biosynthesis-globo and biosynthesis-isoglobo series, and glycosphingolipid biosynthesis-ganglio series (Fig. 2b2). In addition, we discovered that the IPR annotation of DEPs was primarily enriched with immunoglobulin I-set, immunoglobulin-like fold, immunoglobulin C1-set, cadherin and cadherin-like (Fig. 2c), and subcellular localization of DEPs was mainly related to extra cell protein (41.94%), plasma membrane protein (32.50%), and lysosome protein (5.83%) (Fig. 2d).

Ab-dependent phagocytosis and complement-dependent myelin lysis were observed in MOGAD [33, 34]. In our results, we discovered that a large proportion of proteins (*P* < 0.05 and FC ≥ 1.5 or FC ≤ 0.67) associated with complement and coagulation cascades were upregulated in the MOGAD group. Subsequently, the expression levels of several complements in the serum of children with MOGAD were measured using an ELISA kit and compared with those in HCs. In contrast, downregulated expression levels of serum complements C1r, C2 and C9 were identified using ELISA analysis in the MOGAD group compared with the control group (Fig. 2e1–4).

### DEPs and dysregulated biologic processes in CSF from patients with MOGAD compared with patients with IND

We also performed proteomics analysis on the CSF of the MOGAD group compared with the MOG-Ab-seronegative and mNGS-negative IND groups. Proteomic profile changes and O-PLS-DA results of CSF proteins from children with MOGAD in comparison to children with IND (MOG Abs and mNGS negative) are presented in Supplementary Fig. 3. At this stage, functional analysis of dysregulated proteins with marked differences between patients with MOGAD and patients with IND, which were identified with *P* < 0.05 and FC ≥ 1.5 or FC ≤ 0.67, are shown in Supplementary Fig. 4.

## Discussion

MOGAD is a CNS demyelinating disease distinct from NMOSDs and multiple sclerosis. MOG Abs from serum samples target full-length, conformationally intact human MOG. Detailed protein data on CSF findings in pediatric patients with MOGAD are lacking. Therefore, we decided to analyze the expression characteristics of proteins in the CSF sample of MOGAD patients to search for potential biomarkers.

Recently, several proteomics studies have been undertaken to identify new CSF biomarkers in Alzheimer’s disease, multiple sclerosis, NMOSD and meningoencephalitis [21, 23–25, 35]. In this study, we performed proteomics analysis to identify biomarkers from CSF samples of MOGAD patients. To the best of our knowledge, this is the first study to determine the CSF protein expression profile from MOGAD children using proteomics analysis. PLS-DA and O-PLS-DA were performed based on proteomic data to evaluate our model fitting and accuracy. We selected five of the top 30 *P* value-ranked proteins that were also in the list of the top 30 proteins with the highest VIP scores, including GGH, CTSF, ITIH5, LTBP4 and LAIR1. Dysregulation of folic acid was likely involved in some diseases, including immune dysfunction [36]. Autopsy of patients with leukemia indicated that folate antagonism might contribute to the development of myelopathy [37]. It is possible to hypothesize that dysregulated GGH expression has the potential to impact MOGAD patients. The human cathepsin family has 11 cysteine proteases, including CTSF. Recent evidence has suggested that cathepsins are involved in modulatory functions by limited proteolysis of proteins using cathepsin gene knockout mice. CTSF is capable of processing invariant chains related to the major histocompatibility complex (MHC) class II and regulating MHC class II antigen presentation as an effector to regulate the immune system [38]. One study suggested that increased expression of cathepsin B, belonging to the cathepsin family, might lead to demyelination in patients with multiple sclerosis [39], while there was no evidence for CTSF influence on myelination thus far. We await future efforts exploring the role of CTSF in MOGAD processes. No studies have reported the underlying molecular mechanisms of ITIH5, LTBP4 and LAIR1 involved in neuroimmunity or myelination.

In our research, we found that complements, including complement components C2, C5, and C9 and complement subcomponent C1r, were upregulated in CSF samples of MOGAD patients, consistent with previous results, indicating that perivascular deposits of activated complements were observed in MOG Ab-associated demyelinating lesions [40, 41]. The complement system is activated through the classical pathway, the lectin pathway (LP), and the alternative pathway. Ficolin-3 was associated with LP [42]. In our study, the expression level of ficolin-3 was increased significantly in CSF samples from MOGAD patients. The inflammatory glycoprotein chitinase-3-like protein 1 (YKL-40) was found to be expressed by microglia and astrocytes [43, 44], and we observed that the expression level of YKL-40 was elevated in CSF samples from MOGAD children. Based on the significantly increased expression level of complements C1r, C2, C5, C6 and C9 in CSF samples from patients with MOGAD compared with HCs, the complements C1r, C2, C5 and C9 levels in serum from participants were further evaluated using an ELISA kit. Although a significant difference in complement expression levels was identified between the two groups, the results showed decreased expression levels of complements C1r, C2 and C9 in serum from patients with MOGAD compared with HCs. Thus, in consideration of the low disease incidence, a multicenter study in the future is needed to obtain large numbers of CSF and serum samples to further certify complement expression levels in patients with MOGAD.

Compared with HCs, MOGAD patients demonstrated significant downregulation of many proteins relevant to neuronal cell adhesion activities, axon guidance, and glycosphingolipid biosynthesis, including CNTN2, NRXN2, NRXN1, CADM3, CDH15, JAM2, NRXN3, NCAM2, CNTN1, NCAM1, CDH2, NACAM, PLXNB2, SEMA6D, SEMA7A, EPHA7, SEMA6A and beta-hexosaminidase. Recent advances suggesting a better understanding of the cellular interactions involved in damage to the axon-oligodendrocyte-myelin unit are important for studying myelin pathology, and myelin dysfunction must be understood in the broader context of nervous system pathophysiology [45]. The expression levels of PLXNB2, SEMA6D, SEMA7A, SEMA6A, and EPHA7, which are relevant for axon guidance, were decreased in our study. Myelin is enriched in glycosphingolipids, and lipid molecules play an important role in generating myelin. Our data revealed that the expression level of beta-hexosaminidase associated with glycosphingolipid biosynthesis was decreased. Pathway analysis revealed a reduction in the cAMP signaling pathway in MOGAG patients. We observed that the expression level of MOG protein is decreased in the CSF of MOGAD patients.

Compared with patients with MOG seronegative and mNGS-negative IND, a range of proteins with dysregulated expression levels were also identified in CSF samples from patients with MOGAD in our study. Tuftelin-interacting protein 11, overlapping between the top 30 DEPs with top-ranked *P* value and FC and top 30 proteins in O-PLS-DA VIP lists, was reported to localize in speckled nuclear domains [46]. The evidence suggested that this protein participated in cellular activity related to RNA splicing. Our results provide novel molecular biomarker candidates for the discrimination of MOGAD from other MOG-seronegative neuroimmune-related disorders and help us further understand the underlying mechanism of the MOGAD process.

Even though our work sets up the foundation for biomarker development on MOGAD patients, our study is still preliminary. The study cohort in our research was relatively small, and the CSF samples were not enough for further verification of these potential biomarkers. The small sample size may lead to overfitting potential compared to the number of candidate protein biomarkers. We will expand the sample size to verify the selection of more specific marker factors in the future. Improvements in understanding the MOGAD mechanisms that lead to relapses are key to improving outcomes in MOGAD patients. Therefore, prospective studies are needed to identify disease-specific biomarkers of results and treatment response [4]. The children with MOGAD in this cohort will be followed up for two years or more. Notably, the CSF samples in this study were all collected before treatment without an impact on proteomics profiling by drugs. We will track the sequelae (remission or relapse) of these patients at least two years later. The potential predictive biomarker will be identified in the next study based on the analysis of DEPs between the remission group and relapse group. Prognostic biomarkers are important in predicting the progression of disease and might guide therapeutic strategies.

### Supplementary Information

Below is the link to the electronic supplementary material.

Supplementary file 1 (PDF 1199 KB)

Supplementary file 2 (PDF 54 KB)

## Data Availability

The datasets generated during the current study are available in the iProX reservoir, which can be accessed using the following link: https://www.iprox.org/page/PSV023.html;?url=1586159341973v65g (password: qNLe).
