# Supplementary material for: Proteomic profiling of cerebrospinal fluid in pediatric myelin oligodendrocyte glycoprotein antibody-associated disease
Source: World J Pediatr. 2022 Dec 12;20(3):259–71. doi: 10.1007/s12519-022-00661-y (PMC10957615; doi:10.1007/s12519-022-00661-y)
Supplement: Supplementary file 1 — (PDF 1199 KB) [file 12519_2022_661_MOESM1_ESM.pdf]

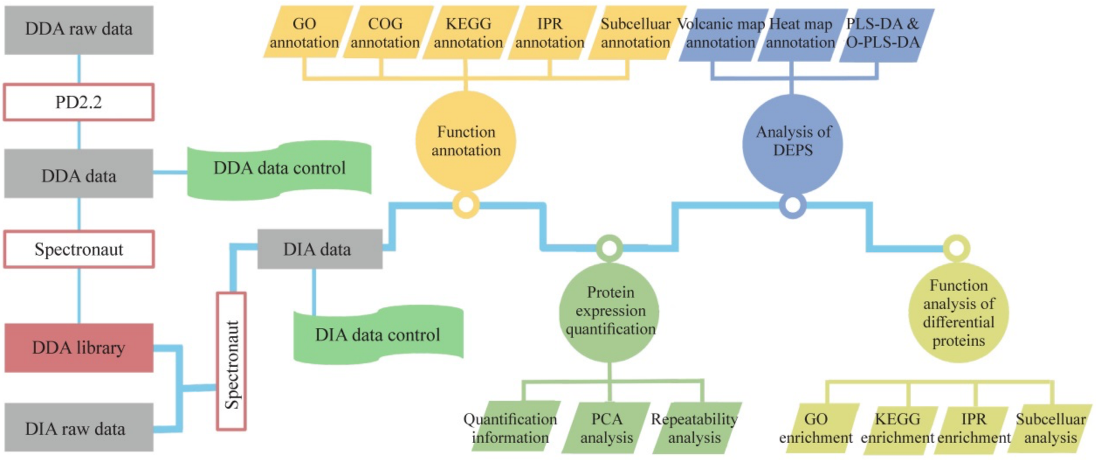


**Supplementary Fig. 1** The flowchart of the study methods. *DDA* data-dependent acquisition, *DIA* data-independent acquisition, *GO* gene ontology, *COG* Clusters of Orthologous Genes, *KEGG* Kyoto Encyclopedia Genes and Genomes, *IPR* InterPro, *PLS-DA* partial least squares discriminant analysis, *O-PLS-DA* orthogonal partial least squares discriminant analysis, *DEPS* differentially expressed proteins, *PCA* principal component analysis

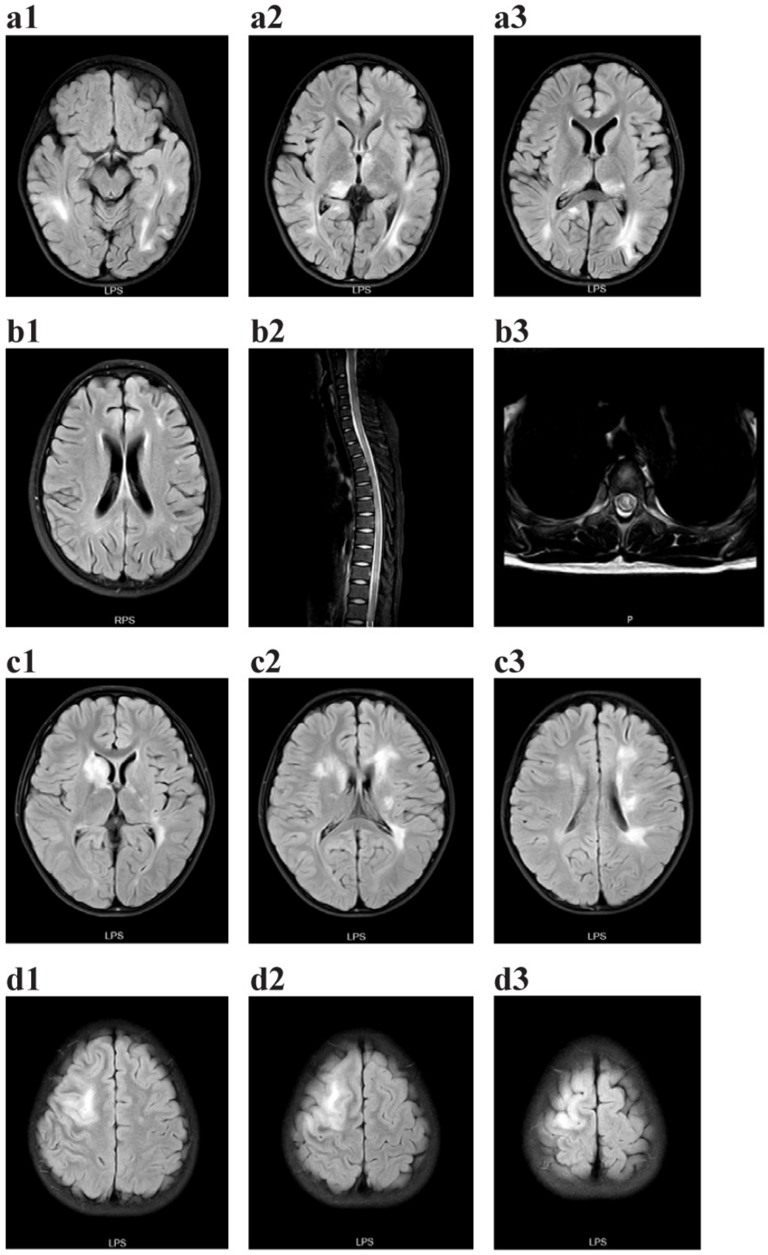


**Supplementary Fig. 2** Representative MRI features in four patients. **a** Patient 1: boy, aged 5.8 years, with clinical-radiological presentation of ADEM including bilateral cerebral hemisphere and thalamus lesions; **b** patient 6: girl, aged 12.9 years, with clinical-radiological presentation of ADEM including multifocal white matter lesion and longitudinally extensive spinal cord involvement; **c** patient 8: girl, aged 13.2 years, with clinical-radiological presentation of EN including right frontal lobe lesion; **d** patient 10: boy, aged 8.8 years, with clinical-radiological presentation of ADEM including diffuse asymmetric white matter lesion. *ADEM* acute disseminated demyelinating syndromes, *EN* encephalitis, *MRI* magnetic resonance imaging

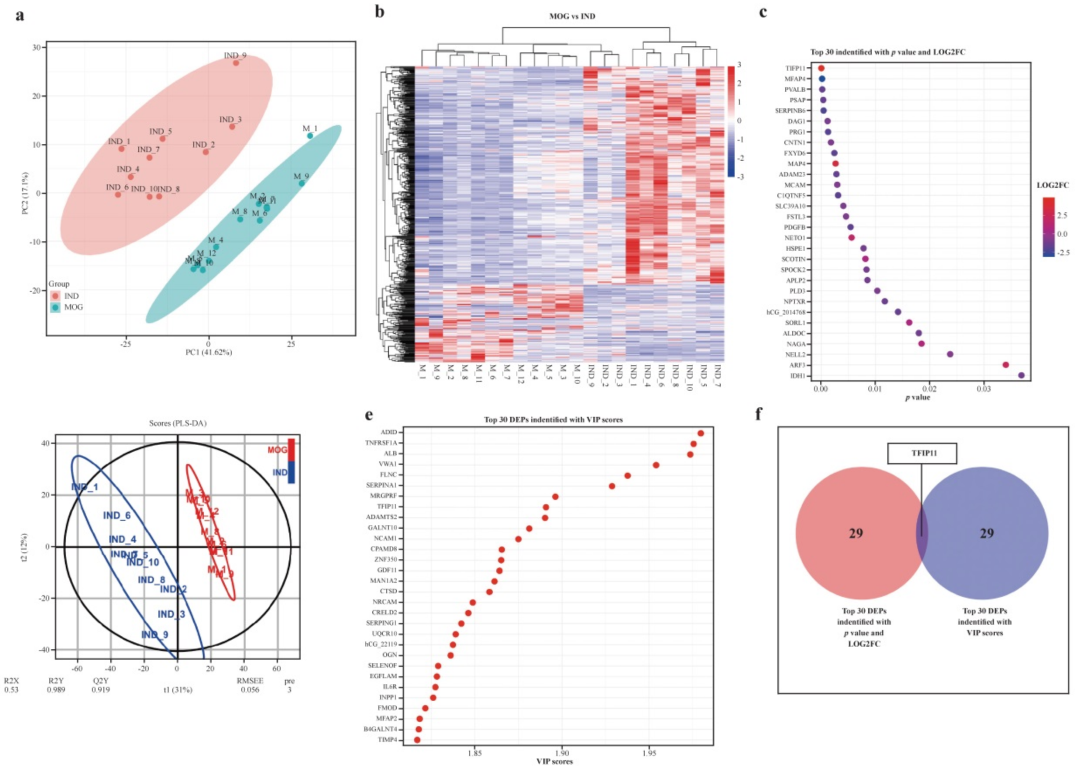


**Supplementary Fig. 3** Characteristics of DEPs between children with MOGAD and IND. **a** PCA on whole proteins from proteomics data; **b** the heatmap of cluster analysis based on MS results; **c** top 30 DEPs identified with *P* value and log2FC; **d** O-PLS-DA of DEPs between children with MOGAD and healthy children; **e** top 30 DEPs identified with most importance using O-PLS-DA; **f** five DEPs selected with top-ranked *P* value, FC and VIP scores. *DEPs* differentially expressed proteins, *MOGAD* myelin oligodendrocyte glycoprotein antibody associated disease, *IND* MOG-seronegative and metagenomics next generation sequencing-negative inflammatory neurological diseases, *PCA* principal component analysis, *MS* mass spectrometry, *O-PLS-DA* orthogonal partial least squares discriminant analysis, *FC* fold change, *VIP* variable importance projection

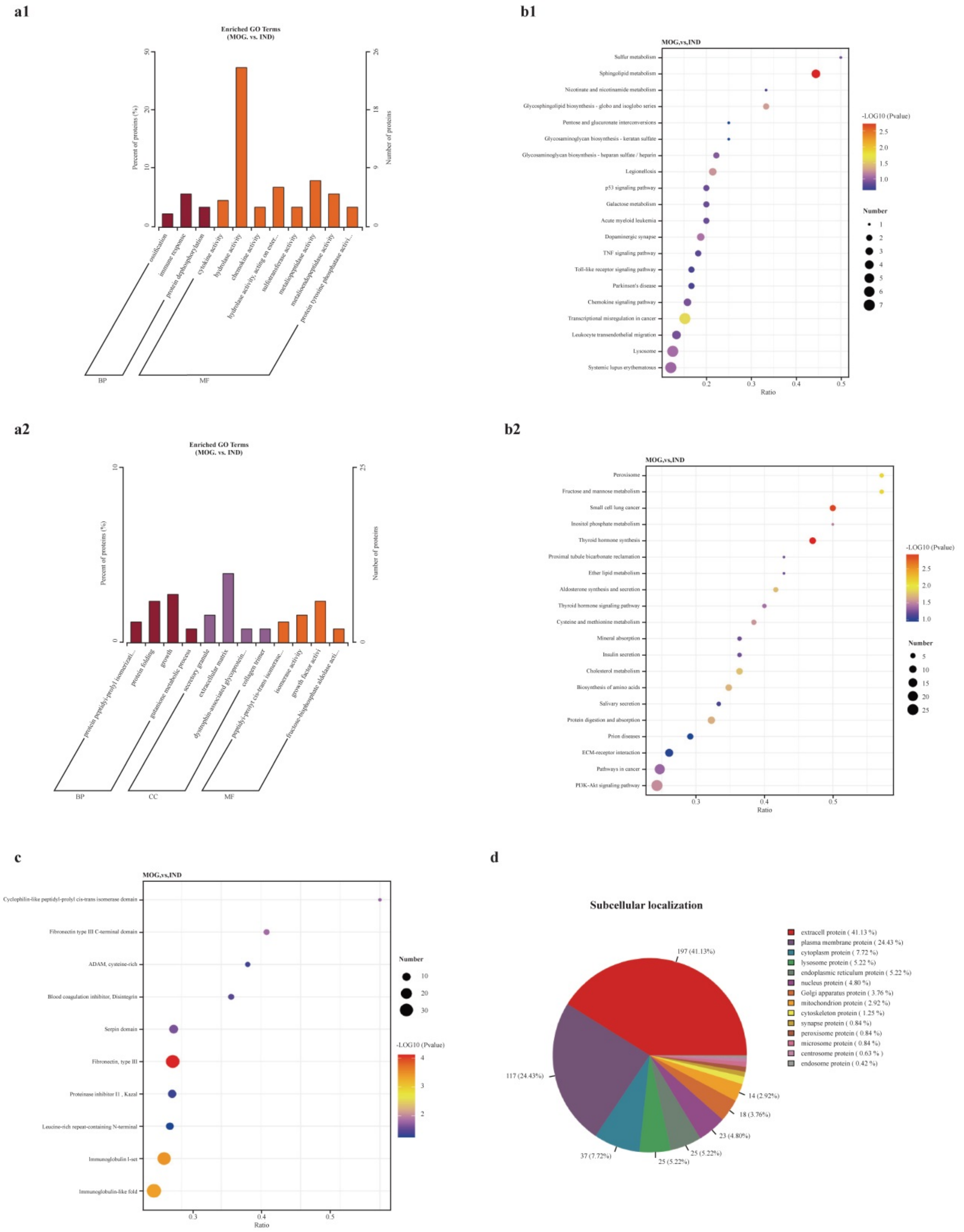


**Supplementary Fig. 4** Function analysis of DEPs between children with MOGAD and IND. **a** GO terms analysis on upregulated (**a1**) and downregulated proteins (**a2**) in MOGAD children compared with IND children are listed with lengths of the bars representing the numbers of related DEPs as labelled at the bottom; **b** the top 20 KEGG analysis for upregulated (**b1**) and downregulated (**b2**) are demonstrated pathways with the size of dots representing the numbers of related DEPs displayed at the bottom; **c**

IPR analysis of DEPs: distribution of the DEPs in MOGAD versus IND comparison with IPR annotation; **d** functional classification by subcellular localization. GO terms and enrichment pathways were in accordance with the following rules: *P* value. *DEPs* differentially expressed proteins, *MOGAD* myelin oligodendrocyte glycoprotein antibody associated disease, *IND* MOG-seronegative and metagenomics next generation sequencing-negative inflammatory neurological diseases, *GO* gene ontology, *KEGG* Kyoto Encyclopedia Genes and Genomes, *IPR* InterPro, *FDR* false discovery rate, *TNF* tumor necrosis factor, *ECM* extracellular matrix, *ADAM* a disintegrin and metalloproteinase, *BP* biological process, *CC* cellular component, *MF* molecular function
