# Supplementary material for: Proteomic profiling of cerebrospinal fluid in pediatric myelin oligodendrocyte glycoprotein antibody-associated disease
Source: World J Pediatr. 2022 Dec 12;20(3):259–71. doi: 10.1007/s12519-022-00661-y (PMC10957615; doi:10.1007/s12519-022-00661-y)
Supplement: Supplementary file 2 — (PDF 54 KB) [file 12519_2022_661_MOESM2_ESM.pdf]

**Supplementary Table 1.** Peptide fraction separation liquid chromatography elution gradient

| Time (min) | Flow rate (mL/min) | Mobile phase (A), % | Mobile phase (B), % |
|---|---|---|---|
| 0 | 1 | 97 | 3 |
| 10 | 1 | 95 | 5 |
| 20 | 1 | 80 | 20 |
| 27 | 1 | 60 | 40 |
| 29 | 1 | 50 | 50 |
| 30 | 1 | 30 | 70 |
| 35 | 1 | 0 | 100 |

**Supplementary Table 2.** Liquid chromatography elution gradient

| Time (min) | Flow rate (nL/min) | Mobile phase (A), % | Mobile phase (B), % |
|---|---|---|---|
| 0 | 600 | 95 | 5 |
| 1 | 600 | 92 | 8 |
| 76 | 600 | 70 | 30 |
| 81 | 600 | 50 | 50 |
| 82 | 600 | 5 | 95 |
| 92 | 600 | 5 | 95 |
| 92.5 | 600 | 95 | 5 |
| 93.5 | 600 | 95 | 5 |
| 94.5 | 600 | 5 | 95 |
| 99 | 600 | 5 | 95 |
| 100 | 600 | 95 | 5 |

**Supplementary Table 3.** Data-independent acquisition mode scan window information list

| Width (m/z) | Start (m/z) | End (m/z) | Median (m/z) |
|---|---|---|---|
| 13 | 400 | 413 | 406.5 |
| 12 | 413 | 425 | 419.0 |
| 12 | 425 | 437 | 431.0 |
| 11 | 437 | 448 | 442.5 |
| 11 | 448 | 459 | 453.5 |
| 10 | 459 | 469 | 464.0 |
| 10 | 469 | 479 | 474.0 |
| 9 | 479 | 488 | 483.5 |
| 9 | 488 | 497 | 492.5 |
| 10 | 497 | 507 | 502.0 |
| 9 | 507 | 516 | 511.5 |
| 9 | 516 | 525 | 520.5 |
| 9 | 525 | 534 | 529.5 |
| 9 | 534 | 543 | 538.5 |
| 9 | 543 | 552 | 547.5 |
| 9 | 552 | 561 | 556.5 |
| 9 | 561 | 570 | 565.5 |
| 9 | 570 | 579 | 574.5 |
| 9 | 579 | 588 | 583.5 |
| 9 | 588 | 597 | 592.5 |
| 9 | 597 | 606 | 601.5 |
| 9 | 606 | 615 | 610.5 |
| 9 | 615 | 624 | 619.5 |
| 9 | 624 | 633 | 628.5 |
| 9 | 633 | 642 | 637.5 |
| 10 | 642 | 652 | 647.0 |
| 9 | 652 | 661 | 656.5 |
| 8 | 661 | 669 | 665.0 |
| 10 | 669 | 679 | 674.0 |
| 10 | 679 | 689 | 684.0 |
| 10 | 689 | 699 | 694.0 |
| 10 | 699 | 709 | 704.0 |
| 10 | 709 | 719 | 714.0 |
| 11 | 719 | 730 | 724.5 |
| 11 | 730 | 741 | 735.5 |
| 11 | 741 | 752 | 746.5 |
| 11 | 752 | 763 | 757.5 |
| 11 | 763 | 774 | 768.5 |
| 12 | 774 | 786 | 780.0 |

| | | | |
|---|---|---|---|
| 15 | 786 | 801 | 793.5 |
| 15 | 801 | 816 | 808.5 |
| 16 | 816 | 832 | 824.0 |
| 15 | 832 | 847 | 839.5 |
| 15 | 847 | 862 | 854.5 |
| 19 | 862 | 881 | 871.5 |
| 20 | 881 | 901 | 891.0 |
| 23 | 901 | 924 | 912.5 |
| 23 | 924 | 947 | 935.5 |
| 26 | 947 | 973 | 960.0 |
| 31 | 973 | 1004 | 988.5 |
